# Biophysical Basis for Three Distinct Dynamical Mechanisms of Action Potential Initiation

**DOI:** 10.1371/journal.pcbi.1000198

**Published:** 2008-10-10

**Authors:** Steven A. Prescott, Yves De Koninck, Terrence J. Sejnowski

**Affiliations:** 1 Computational Neurobiology Laboratory, Salk Institute, La Jolla, California, United States of America; 2 Howard Hughes Medical Institute, La Jolla, California, United States of America; 3 Division de Neurobiologie Cellulaire, Centre de Recherche Université Laval Robert-Giffard, Québec, Québec, Canada; 4 Division of Biological Sciences, University of California San Diego, La Jolla, California, United States of America; UFR Biomédicale de l'Université René Descartes, France

## Abstract

Transduction of graded synaptic input into trains of all-or-none action potentials (spikes) is a crucial step in neural coding. Hodgkin identified three classes of neurons with qualitatively different analog-to-digital transduction properties. Despite widespread use of this classification scheme, a generalizable explanation of its biophysical basis has not been described. We recorded from spinal sensory neurons representing each class and reproduced their transduction properties in a minimal model. With phase plane and bifurcation analysis, each class of excitability was shown to derive from distinct spike initiating dynamics. Excitability could be converted between all three classes by varying single parameters; moreover, several parameters, when varied one at a time, had functionally equivalent effects on excitability. From this, we conclude that the spike-initiating dynamics associated with each of Hodgkin's classes represent different outcomes in a nonlinear competition between oppositely directed, kinetically mismatched currents. Class 1 excitability occurs through a saddle node on invariant circle bifurcation when net current at perithreshold potentials is inward (depolarizing) at steady state. Class 2 excitability occurs through a Hopf bifurcation when, despite net current being outward (hyperpolarizing) at steady state, spike initiation occurs because inward current activates faster than outward current. Class 3 excitability occurs through a quasi-separatrix crossing when fast-activating inward current overpowers slow-activating outward current during a stimulus transient, although slow-activating outward current dominates during constant stimulation. Experiments confirmed that different classes of spinal lamina I neurons express the subthreshold currents predicted by our simulations and, further, that those currents are necessary for the excitability in each cell class. Thus, our results demonstrate that all three classes of excitability arise from a continuum in the direction and magnitude of subthreshold currents. Through detailed analysis of the spike-initiating process, we have explained a fundamental link between biophysical properties and qualitative differences in how neurons encode sensory input.

## Author Summary

Information is transmitted through the nervous system in the form of action potentials or spikes. Contrary to popular belief, a spike is not generated instantaneously when membrane potential crosses some preordained threshold. In fact, different neurons employ different rules to determine when and why they spike. These different rules translate into diverse spiking patterns that have been observed experimentally and replicated time and again in computational models. In this study, our aim was not simply to replicate different spiking patterns; instead, we sought to provide deeper insight into the connection between biophysics and neural coding by relating each to the process of spike initiation. We show that Hodgkin's three classes of excitability result from a nonlinear competition between oppositely directed, kinetically mismatched currents; the outcome of that competition is manifested as dynamically distinct spike-initiating mechanisms. Our results highlight the benefits of forward engineering minimal models capable of reproducing phenomena of interest and then dissecting those models in order to identify general explanations of how those phenomena arise. Furthermore, understanding nonlinear dynamical processes such as spike initiation is crucial for definitively explaining how biophysical properties impact neural coding.

## Introduction

Action potentials, or spikes, are responsible for transmitting information through the nervous system [1]. The biophysical basis of spike generation is well established [2], but the stereotypic spike shape belies variation in spike initiating mechanisms. The myriad different ion channels expressed in different neurons produce diverse patterns of repetitive spiking [3],[4]. The fact that equivalent stimulation can elicit qualitatively different spiking patterns in different neurons attests that intrinsic coding properties differ significantly from one neuron to the next.

Hodgkin recognized this and identified three basic classes of neurons distinguished by their frequency-current (*f–I*) curves [5]. The ability of class 1 neurons to fire slowly in response to weak stimulation endows them with a continuous *f–I* curve, whereas class 2 neurons have a discontinuous *f–I* curve because of their inability to maintain spiking below a critical frequency. Class 3 neurons fail to spike repetitively, typically spiking only once at the onset of stimulation; their *f–I* curve is undefined since calculation of firing rate requires at least two spikes for an interspike interval (ISI) to be measured. Although neuronal coding properties may change on slow time scales (e.g., because of adaptation or bursting), Hodgkin's classification provides a fundamental description of analog-to-digital transduction occurring on the time scale of single ISIs, and therefore addresses the very essence of how individual spikes are initiated.

The distinction between class 1 and 2 excitability has proven extremely useful for distinguishing neurons with different coding properties [6]–[12]. Properties such as the phase-reset curve are not directly related to the *f–I* curve per se, but can be explained by the same dynamical mechanisms that account for continuity or discontinuity of the *f–I* curve. In terms of their nonlinear dynamics, class 1 neurons spike repetitively when their stable fixed point is destroyed through a saddle-node on invariant circle (SNIC) bifurcation (sometimes referred to simply as a saddle-node bifurcation) whereas class 2 neurons spike repetitively when their fixed point is destabilized through a subcritical Hopf bifurcation [7],[13]. The dynamical mechanism for spike initiation in class 3 neurons has not been explained. Given that mechanistic understanding of spike initiation clearly provides greater insight into neural coding than a purely phenomenological description of spiking pattern, the coding properties of class 3 neurons could be more readily explained if we understood the spike initiating dynamics in those neurons. Furthermore, abstract dynamical explanations of spike initiation must be translated into biophysically interpretable mechanisms if we are to explain the biophysical basis of neural coding.

This study set out to identify the biophysical basis for qualitative differences in neural coding exemplified by Hodgkin's three classes. By relating spike initiating dynamics with transduction properties, and by identifying the biophysical basis for those dynamics, we explain how Hodgkin's three classes of excitability result from a nonlinear, time-dependent competition between oppositely directed currents.

## Results

### Reproduction of Experimental Data in a Two-Dimensional Model

**Figure 1:**
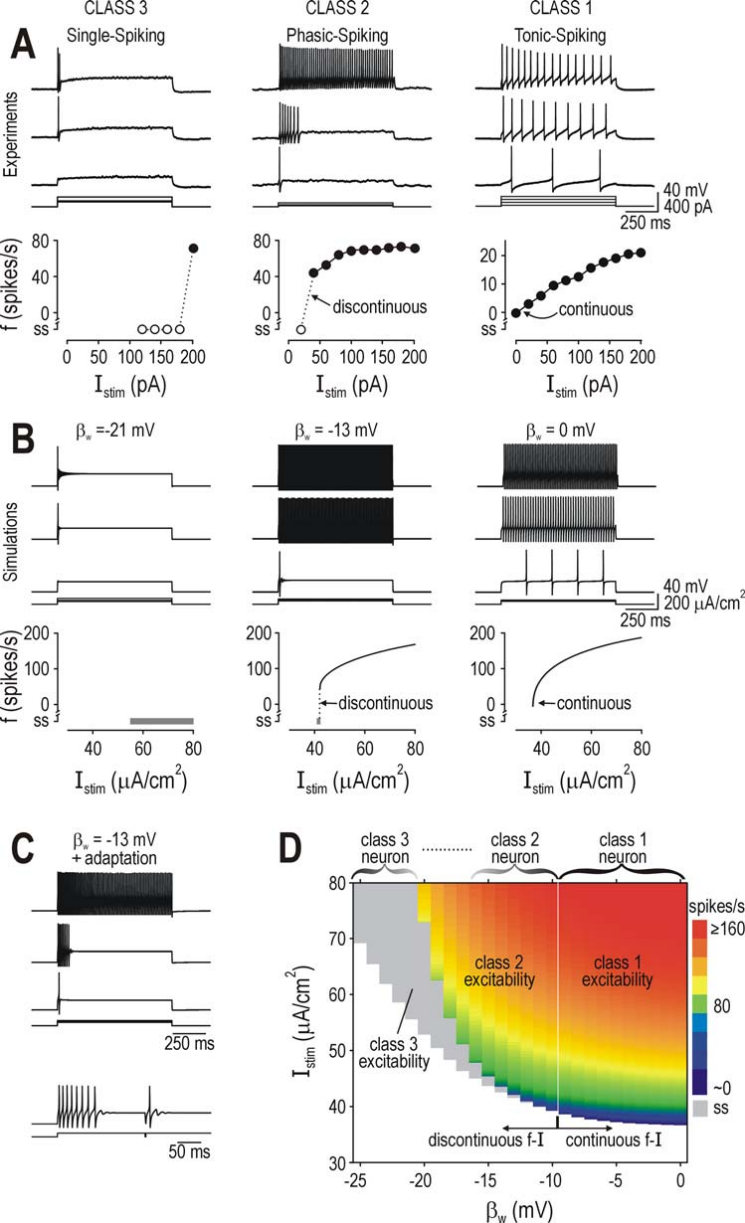
Hodgkin's three classes of neuronal excitability. (A) Sample responses from spinal lamina I neurons representing each of Hodgkin's three classes. Hodgkin's classification is based on the *f–I* curve which is continuous (class 1), discontinuous (class 2), or undefined because measurement of firing rate requires at least two spikes (class 3). Data points comprising a single spike (*ss*) are indicated with open symbols in (A) or gray shading in (B–D). (B) Each cell class could be reproduced in a Morris-Lecar model by varying a single parameter, in this case *β*_w_. Like in (A), rheobasic stimulation (minimum *I*_stim_ eliciting ≥1 spike) elicited a single spike at short latency in class 2 and 3 neurons compared with slow repetitive spiking in class 1 neurons. Despite reproducing the discontinuous *f–I* curve, the 2D model could not reproduce the phasic-spiking pattern. (C) Phasic-spiking was generated by adding slow adaptation, thus giving a 3D model described by *C dV*/*dt* = *I*_stim_−*g*¯_fast_ *m*_∞_(*V*)(*V*−*E*_Na_)−*g*¯_slow_ *w*(*V*−*E*_K_)−*g*_leak_(*V*−*E*_leak_)−*g*_adapt_ *a*(*V*−*E*_K_) and 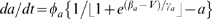 where *a* controls activation of adaptation and *g*¯_adapt_ = 0.5 mS/cm^2^, *φ*_a_ = 0.05 ms^−1^, *β*_a_ = −40 mV, and *γ*_a_ = 10 mV. Bottom traces show single-spike elicited by second stimulus applied shortly after the end of first stimulus, which suggests that adaptation slowly shifts the neuron from class 2 towards class 3 excitability. (D) Firing rate (color) is plotted against *I*_stim_ and *β*_w_. Separable regions of the graph correspond to different classes of excitability. Neuronal classification is based on which class of excitability is predominant (i.e., exhibited over the broadest range of *I*_stim_) and is indicated above the graph.

Spinal sensory neurons fall into several categories based on spiking pattern [14]–[18]. Tonic-, phasic-, and single-spiking lamina I neurons exhibit the characteristic features of class 1, 2, and 3 excitability, respectively, based on their *f–I* curves (Figure 1A). Spiking pattern is related to, but not synonymous with, Hodgkin's classification scheme. For instance, phasic-spiking neurons are not class 2 because they stop spiking before the end of stimulation, but the fact that they stop spiking so abruptly suggests that they cannot maintain spiking below a certain rate, which is consistent with a discontinuous (class 2) *f–I* curve; in contrast, adaptation causes tonic-spiking neurons to spike more slowly but without stopping, consistent with a continuous (class 1) *f–I* curve.

To explain the differences between cell classes, our first step was to reproduce the behavior of each class in as simple a computational model as possible, and then to analyze that minimal model. We found that a 2D Morris-Lecar-like model could display class 1, 2, or 3 excitability with variation of as few as one parameter (Figure 1B). The discontinuous *f–I* curve characteristic of class 2 excitability was observed when parameter *β*_w_ (see below) was set between values giving class 1 or 3 excitability, but phasic-spiking could not be reproduced in the 2D model. Phasic-spiking was achieved by incorporating adaptation (Figure 1C), although this makes the model 3D because adaptation operates on a slower time scale than the activation and recovery variables comprising the 2D model. If the neuron was allowed to fully adapt before a second stimulus was applied, the second stimulus elicited only one spike, which suggests that adaptation caused a slow transition from class 2 to class 3 excitability (see below). But, since un-adapted phasic-spiking neurons exhibit class 2 excitability, we used the class 2 model without adaptation for subsequent phase plane analysis.

In the process of building the model (see Methods), *β*_w_ was identified as an important parameter given its capacity to convert the model between all three classes of excitability. The biophysical meaning of *β*_w_ is deferred until Figure 4, after its functional significance has been established. See Figure 8 for the effects of changing other parameters. Therefore, to begin, we explored the effects on the model's *f–I* curve of systematically varying *β*_w_ (Figure 1D). The model exhibited class 1 excitability for *β*_w_>−10 mV, but class 2 and 3 excitability coexisted for all *β*_w_<−10 mV; in other words, class 2 or 3 excitability was exhibited depending on stimulus intensity *I*_stim_. This is evident in Figure 1B where, in the model with *β*_w_ = −13 mV, rheobasic stimulation elicited a single spike while stronger stimulation elicited repetitive spiking. This pattern is characteristic of phasic-spiking spinal lamina I neurons (Figure 1A) and is commonly observed in other “class 2” neurons including the squid giant axon [2], trigeminal motoneurons [19], and fast-spiking neocortical interneurons [10],[20]. Conversely, “class 3” neurons should theoretically begin spiking repetitively if given extremely strong stimulation. In reality, strong stimulation elicits, at most, a burst of 2–4 high frequency spikes in single-spiking spinal lamina I neurons [14], which is consistent with Hodgkin's classification in which class 3 neurons are said to “repeat *only with difficulty* or not at all” [5]. Responses to strong stimulation can be more accurately reproduced in the model by incorporating slow processes like cumulative Na^+^ channel inactivation, but such processes were not included in the models analyzed here in order to keep the model as simple as possible and because such strong stimulation is arguably unphysiological in the first place.

Thus, neurons should not strictly be labeled class 2 or 3 but, rather, as exhibiting predominantly class 2 or 3 excitability based on the range of *I*_stim_ over which they exhibit each class. However, phasic-spiking lamina I neurons exhibited class 3 excitability over a negligible stimulus range (i.e., <5% of the range for *I*_stim_ tested as high as 200 pA) and single-spiking neurons never exhibited class 2 excitability for *I*_stim_ as high as 500 pA. So, although a neuron may exhibit both class 2 and class 3 excitability, lamina I neurons exhibit *almost entirely* one or the other class over the physiologically relevant stimulus range. We therefore label tonic-, phasic-, and single-spiking spinal lamina I neurons as class 1, 2, and 3 neurons, respectively. Although practical, such labeling may be inappropriate for other cell populations if a more balanced mix of class 2 and 3 excitability exists within a single cell type.

### Dynamical Basis for Different Classes of Excitability

Having reproduced each class of excitability in a 2D model, our next step was to exploit the simplicity of that model to uncover the spike initiating dynamics associated with each class. Because the model is 2D, its behavior can be explained entirely by the interaction between two variables: a fast activation variable *V* and a slower recovery variable *w*. This interaction can be visualized by plotting *V* against *w* to create a phase portrait. Behavior of the model can be understood by how the *V*- and *w*-nullclines intersect; nullclines represent everywhere in phase space where *V* or *w* remain constant.

**Figure 2:**
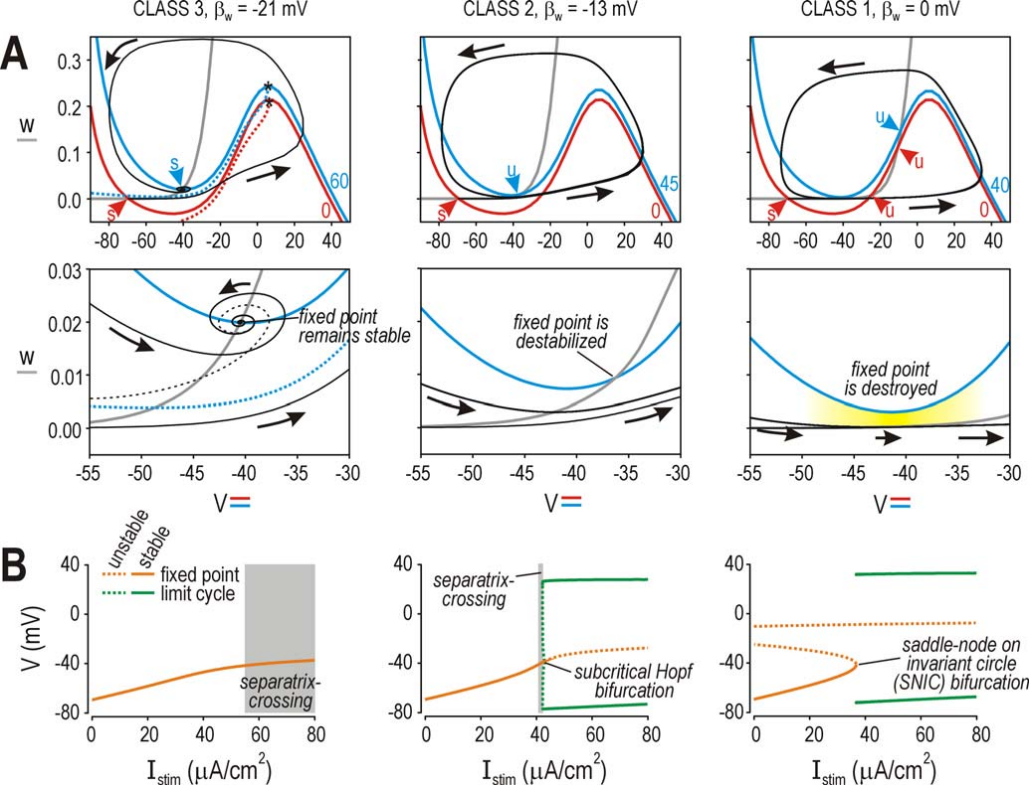
Each class of excitability is derived from a distinct dynamical mechanism of spike initiation. (A) Phase planes show the fast activation variable *V* plotted against the slower recovery variable *w*. Nullclines represent all points in phase space where *V* or *w* remain constant. *V*-nullclines (colored) were calculated at rest (red) and at the onset of stimulation (blue) (*I*_stim_ is indicated beside each curve); *w*-nullclines do not change upon stimulation and are plotted only once (gray). Black curves show response of model with direction of trajectory indicated by arrows. Class 1 neuron: Red and gray nullclines intersect at three points (red arrowheads) representing stable (*s*) or unstable (*u*) fixed points. Stimulation shifts that *V*-nullcline upward and destroys two of those points, thereby allowing the system to enter a limit cycle and spike repetitively. The trajectory slows as it passes through constriction between blue and gray nullclines (yellow shading) thereby allowing the neuron to spike slowly, hence the continuous *f–I* curve. Class 2 neuron: Red and gray curves intersect at a single, stable fixed point. Spiking begins when stimulation destabilizes (rather than destroys) that point. The *f–I* curve is discontinuous because slow spiking is not possible without the constriction (compare with class 1 neuron). Class 3 neuron: Stimulation displaces but does not destroy or destabilize the fixed point. System variables *V*,*w* can follow different paths to the newly positioned fixed point: a single spike is initiated when stimulation instantaneously displaces the quasi-separatrix (dotted curves) so that the system, which existed above the (red) quasi-separatrix prior to stimulation, finds itself below the (blue) quasi-separatrix once stimulation begins; the trajectory must go around the head of the quasi-separatrix (*) to get to the new fixed point – we refer to this mechanism of spike initiation as a quasi-separatrix-crossing or QSC. Dashed black curve shows alternative, subthreshold path that would be followed if trajectory remained above the (blue) quasi-separatrix. (B) Bifurcation diagrams show voltage at fixed point and at max/min of limit cycle as *I*_stim_ is increased. A bifurcation represents the transition from quiescence to repetitive spiking. Type of bifurcation is indicated on each plot. The range of *I*_stim_ over which a QSC occurs is indicated in gray and was determined by separate simulations since a QSC is not revealed by bifurcation analysis.

Right panels of Figure 2A illustrate the spike initiating dynamics associated with class 1 excitability. Before stimulation, the *V*- and *w*-nullclines intersect at three points; the leftmost intersection constitutes a stable fixed point that controls membrane potential. Stimulation shifts the *V*-nullcline upwards. If the *V*-nullcline was shifted far enough (i.e., if *I*_stim_ exceeded rheobase), two of the intersection points were destroyed and the class 1 model began to spike repetitively. Disappearance of the two fixed points and the qualitative change in behavior that results (i.e., the transition from quiescence to repetitive spiking) is referred to most precisely as a saddle-node on invariant circle (SNIC) bifurcation [13].

Center panels of Figure 2A illustrate the spike initiating dynamics associated with class 2 excitability. Before stimulation, the *V*- and *w*-nullclines intersect at a single, stable point. Spiking began when the vertical shift in the *V*-nullcline caused by stimulation destabilized that point through a Hopf bifurcation [7]. Destabilization occurred when the *V*- and *w*-nullclines intersected to the right of the local minimum in the *V*-nullcline whereas the fixed point was stable when the nullclines intersected to the left of the minimum (this is not strictly true, but it is a very close approximation). Destabilization of the fixed point (in class 2 excitability) is distinct from destruction of the stable fixed point (in class 1 excitability), but both cause the neuron to spike repetitively rather than remaining at a stable, subthreshold voltage.

Left panels of Figure 2A illustrate the spike initiating dynamics associated with class 3 excitability. In this case, the *V*- and *w*-nullclines intersected at a single, stable point that remained stable for *I*_stim_>rheobase, meaning spike initiation occurred without a bifurcation. The system moved to the *newly positioned* stable fixed point, but could do so via different paths: trajectories starting below the quasi-separatrix made a long excursion around the elbow of the *V*-nullcline, resulting in a single spike; trajectories starting above the quasi-separatrix followed a more direct, subthreshold route. We refer to the boundary in phase space from which trajectories diverge (see Figure 1 in [21]) as a quasi-separatrix (see below). Importantly, although system variables *V* and *w* cannot change instantaneously, the quasi-separatrix does move instantaneously as *I*_stim_ changes; therefore, stimulation can move the quasi-separatrix so that a point (*V*,*w*) that was above the quasi-separatrix before stimulation ends up below the quasi-separatrix during stimulation. We refer to this mechanism as a quasi-separatrix-crossing (QSC) since spike initiation requires that system variables cross from one side of the quasi-separatrix to the other. Such a mechanism was first described by Fitzhugh [22],[23].

The quasi-separatrix was plotted by integrating backward in time (−0.01 ms time step) from point * indicated on Figure 2A; that point was chosen based on where forward trajectories clinging to the quasi-separatrix eventually dissipate, thus suggesting the end of the quasi-separatrix, which is arguably the best location to begin plotting the reverse trajectory. We refer to this boundary as a quasi-separatrix since a true separatrix corresponds to the stable manifold associated with a saddle-point, but both represent a boundary from which trajectories diverge.

Figure 2B shows the bifurcation diagrams associated with the dynamical mechanisms explained in Figure 2A. The SNIC and Hopf bifurcations are readily distinguishable on those diagrams. Transition between these two types of bifurcations occurred near *β*_w_ = −10 mV, which, on the phase plane, corresponds to the *V*- and *w*-nullclines meeting tangentially at the minimum of the *V*-nullcline so that rheobasic stimulation simultaneously destroys and destabilizes the fixed point (data not shown). There was no bifurcation for *β*_w_ = −21 mV and *I*_stim_<80 µA/cm^2^; stronger stimulation eventually caused a Hopf bifurcation, but such stimulation is unphysiological and is likely to activate other processes not included in the model (see above). The range of *I*_stim_ over which spike initiation occurred through a QSC is indicated with gray shading. Notice that a neuron that generated repetitive spiking through a Hopf bifurcation (*β*_w_ = −13 mV) would, for a narrow range of weaker *I*_stim_, generate a single spike through a QSC, consistent with data in Figure 1.

### Validation of the Model

**Figure 3:**
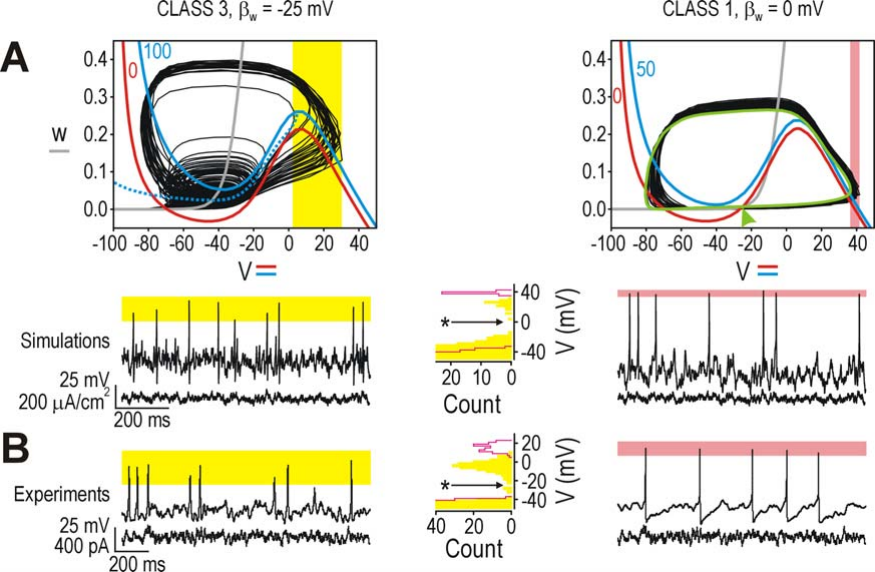
Comparison of spikes initiated through different dynamical mechanisms. (A) Spikes initiated through a QSC or SNIC bifurcation exhibit different spike amplitude variability. Data are from 2D models stimulated with noisy *I*_stim_ (σ_noise_ = 10 µA/cm^2^). *V*-nullclines are shown for rest (red) and for one stimulus intensity (blue) although *I*_stim_ varies continuously during stimulation. Spikes initiated through a QSC exhibit variable amplitudes (yellow shading) because variations in *I*_stim_ affect the *V-w* trajectory: trajectories starting close to the quasi-separatrix (produced by *I*_stim_ fluctuations just exceeding rheobase) produce smaller spikes than trajectories starting further from the quasi-separatrix (produced by larger *I*_stim_ fluctuations). Spikes initiated through an SNIC bifurcation exhibit little variability (pink shading) because all trajectories follow the invariant circle once the heteroclinic trajectories (green curves) fuse at the moment of the SNIC bifurcation to form a single homoclinic orbit. Histogram shows distribution of voltage maxima; maxima above cutoff (*) are considered spikes. Distributions differed significantly between cell classes after normalizing by maximum or by average spike amplitude (*p*<0.005 and *p*<0.001, respectively; Kolmogorov-Smirnov test). (B) As predicted, class 3 (single-spiking) neurons showed significantly greater variability in spike amplitude than class 1 (tonic-spiking) neurons (*p*<0.001 regardless of normalization by peak or average; Kolmogorov-Smirnov test). σ_noise_ = 10 pA.

Since model parameters were chosen to produce one or another spiking pattern, simply reproducing a given pattern is not necessarily informative—this constitutes an inverse problem akin to circular reasoning. To ensure that the model does not simply mimic spiking pattern, it must predict behaviors separate from those used to choose parameters. The model makes such a prediction: spikes initiated through different dynamical mechanisms are predicted to exhibit different variability in their amplitudes. Specifically, spikes initiated through an SNIC bifurcation should have uniform amplitudes because all suprathreshold trajectories follow the invariant circle formed when the stable manifolds (green curves on Figure 3A) fuse at the moment of the bifurcation. In contrast, spikes initiated through a QSC are predicted to have variable amplitudes that are sensitive to stimulus intensity because each trajectory (including how far it extends on the abscissa) depends on where that trajectory starts relative to the quasi-separatrix.

To test this, we stimulated the model neurons with noisy, near-threshold *I*_stim_ fluctuations. As predicted, the class 1 model produced uniformly sized spikes whereas the class 3 model produced variably sized spikes (Figure 3A). The class 2 model exhibited spikes with intermediate variability (data not shown), which was expected given that near-threshold stimulation can elicit spikes through a QSC in this cell class (see above). Equivalent testing in real neurons confirmed the predicted pattern of spike amplitude variability (Figure 3B). Since spike amplitude variability was not the basis for choosing model parameters, experimental confirmation of our prediction of differential variability in spike amplitude argues that our model and the dynamical mechanisms inferred therefrom provide a robust explanation of spike initiation rather than superficially mimicking spiking pattern.

### Biophysical Correlate of the Differences between Class 1, 2, and 3 Models

**Figure 4:**
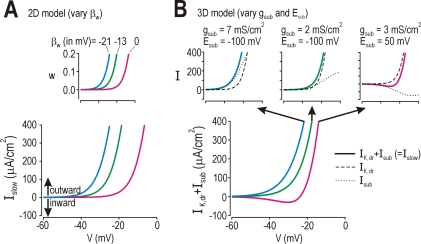
Biophysical correlate of differences in *β*_w_. (A) The *w*-nullcline (inset) corresponds to the voltage-dependent activation curve for *I*_slow_. Horizontal positioning of that curve is controlled by *β*_w_. Differences between class 1, 2, and 3 models may thus reflect differences in the voltage-dependency of *I*_slow_. (B) It is more likely, however, that the components of *I*_slow_ vary between cells of different classes (see Results). *I*_slow_ may comprise multiple currents with similar kinetics. If *I*_slow_ = *I*_K,dr_+*I*_sub_, the position of the net *I–V* curve can be changed in qualitatively the same way as in (A) by changing the direction and magnitude of *I*_sub_ (see insets) without changing the voltage-dependencies of *I*_sub_ (*β*_z_ = −21 mV, *γ*_z_ = 15 mV) or of *I*_K,dr_ (*β*_y_ = −10 mV, *γ*_y_ = 10 mV); voltage-dependencies of *I*_sub_ and *I*_K,dr_ are different, however, with the former being more strongly activated at subthreshold potentials. These results predict that tonic-spiking neurons express a subthreshold inward current and/or that single-spiking neurons express a subthreshold outward current.

With the model thus validated, our next step was to compare class 1, 2 and 3 models to identify differences in parameters that could be related to differences in the biophysical properties of real neurons. As shown in Figure 1, varying *β*_w_ converted the model between all three classes of excitability. *β*_w_ controls horizontal positioning of the *w*-nullcline on the phase plane (Figure 4A, inset), which corresponds to the voltage-dependency of *I*_slow_ in the 2D model (Figure 4A; see Methods). However, since phenotypic diversity is typically attributed to expression of different channels rather than to drastic changes in the voltage-sensitivity of a single channel [24], we hypothesized that different cell classes express different channels. To model this, we split *I*_slow_ into *I*_K,dr_ and *I*_sub_, thus transforming the 2D model into a 3D model. Grouping currents with similar kinetics is a method for reducing dimensionality (e.g., [25]); here, we deliberately ungroup those currents in order to increase the biological realism of the model. The low-dimensional model is better for mathematical analysis, but the higher-dimensional model is arguably better for biological interpretation.

We fixed the voltage-dependencies of *I*_K,dr_ and *I*_sub_, and varied the direction and magnitude of *I*_sub_ in order to represent variable expression levels of a channel carrying inward or outward current. Those changes affected the net slow current (*I*_sub_+*I*_K,dr_) in the 3D model the same way that varying *β*_w_ affected *I*_slow_ in the 2D model (compare Figure 4B with Figure 4A). Note that the voltage-dependency of *I*_sub_ is such that the current activates at subthreshold potentials. These data therefore suggest that spike initiating dynamics may differ between neurons depending on the expression of different slow ionic currents, and, more specifically, that class 3 neurons express a subthreshold outward current and/or class 1 neurons express a subthrehsold inward current, with class 2 neurons expressing intermediate levels of those currents.

**Figure 5:**
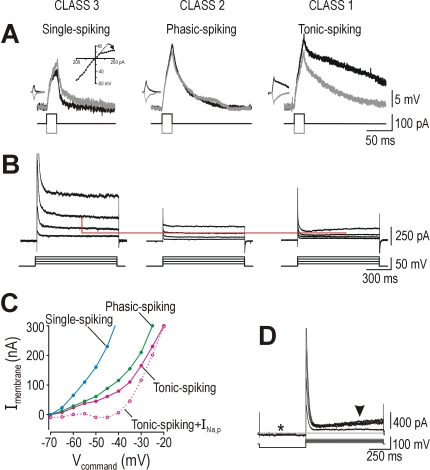
Different classes of spinal lamina I neurons express different subthreshold currents. (A) Traces show responses to 60 pA, 20-ms-long depolarizing pulses (black) and to equivalent hyperpolarizing pulses (gray); the latter are inverted to facilitate comparison with former. In class 1 (tonic-spiking) neurons, depolarization was amplified and prolonged relative to hyperpolarization, consistent with effects of an inward current activated by perithreshold depolarization. Class 3 (single-spiking) neurons exhibited the opposite pattern, consistent with effects of a subthreshold outward current, which is also evident from outward rectification (arrow) in the *I–V* curve. Depolarizing and hyperpolarizing responses were symmetrical in class 2 (phasic-spiking) neurons, consistent with negligible net subthreshold current. (B) Membrane current activated by voltage-clamp steps from −70 mV to −60, −50, −40, and −30 mV. For a given command potential, class 3 neurons exhibited the largest persistent outward current and class 1 neurons exhibited the smallest outward current. Red line highlights difference in current activated by step to −40 mV. (C) Steady-state *I–V* curves for voltage clamp protocols in (B). Because recordings were performed in TTX to prevent unclamped spiking, the persistent Na^+^ current (*I*_Na,p_), which is expressed exclusively in tonic-spiking neurons, was blocked; to correct for this, *I*_Na,p_ measured in separate voltage clamp ramp protocols [26] was added to give a corrected *I–V* curve (dotted line). Compare with Figure 4. (D) 4-AP-sensitive current determined by subtraction of response before and during application of 5 mM 4-AP to a single-spiking neuron. Protocol included prepulse to −100 mV, which revealed a small persistent outward current active at −70 mV that was deactivated by hyperpolarization to −100 mV (*). Although depolarization also activates a large transient outward current, we are concerned with the persistent component (arrowhead); effects of the transient outward current are beyond the scope of this study and were minimized by adjusting pre-stimulus membrane potential to −60 mV for all current clamp protocols. Gray line shows baseline current.

Several lines of experimental evidence support this prediction. First, the response to brief, subthreshold depolarizing pulses was amplified and prolonged relative to the equivalent hyperpolarizing response in class 1 neurons, consistent with effects of a subthreshold inward current (Figure 5A, right); the opposite pattern was observed in class 3 neurons, consistent with effects of a subthrehsold outward current (Figure 5A, left). Second, in voltage clamp, stepping command potential from −70 mV to perithreshold potentials elicited the largest outward current in class 3 neurons, followed by class 2 and class 1 neurons (Figure 5B). The relative positioning of *I–V* curves plotted from those experiments (Figure 5C) bore a striking resemblance to the relative positioning of *I–V* curves in the 2D and 3D models (Figure 4); this is especially true if the persistent Na^+^ current that was blocked by TTX in the aforementioned experiments is taken into account; this current is expressed exclusively in tonic-spiking neurons [26]. Application of 4-AP confirmed the presence of a persistent, low-threshold K^+^ current in single-spiking lamina I neurons (Figure 5D).

### Connection between Subthreshold Currents and Spike-Initiating Dynamics

**Figure 6:**
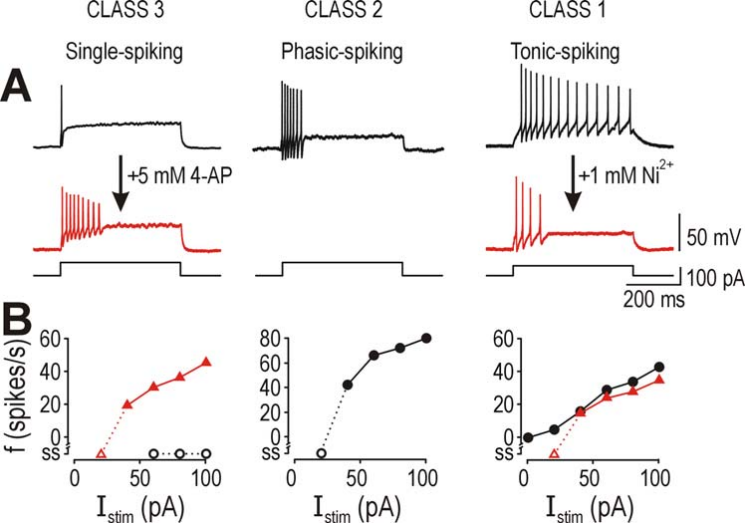
Necessity of oppositely directed subthreshold currents to explain excitability in spinal lamina I neurons. (A) Blocking a subthreshold Ca^2+^ current with Ni^2+^ converted tonic-spiking neurons to phasic-spiking (right). Blocking a subthreshold K^+^ current with 4-AP converted single-spiking neurons to phasic-spiking (left). Compare with naturally occurring phasic-spiking pattern (center). (B) Application of Ni^2+^ and 4-AP converted class 1 and 3 neurons, respectively, to class 2 neurons according to the *f–I* curves. Firing rate was determined from the reciprocal of first interspike interval. According to these data, a subthreshold inward current is necessary for class 1 excitability, a subthreshold outward current is necessary for class 3 excitability, and class 2 excitability occurs when neither current is present.

Thus, lamina I neurons express the sort of currents predicted by our model, but that result is purely correlative, i.e., based on comparison of simulated and experimental *I–V* curves. Are the identified currents necessary and sufficient to explain differences in the excitability of lamina I neurons? One prediction is that blocking the subthreshold inward current in class 1 neurons, or the subthreshold outward current in class 3 neurons, should convert those neurons to class 2 excitability. To test this, we pharmacologically blocked the low-threshold Ca^2+^ or K^+^ current in class 1 and 3 neurons, respectively. As predicted, spiking was converted to a phasic pattern (Figure 6A) and *f–I* curves became discontinuous (Figure 6B) in both cases, consistent with conversion to class 2 excitability. This demonstrates the necessity, in spinal lamina I neurons at least, of subthreshold inward and outward currents for producing class 1 and 3 excitability, respectively.

**Figure 7:**
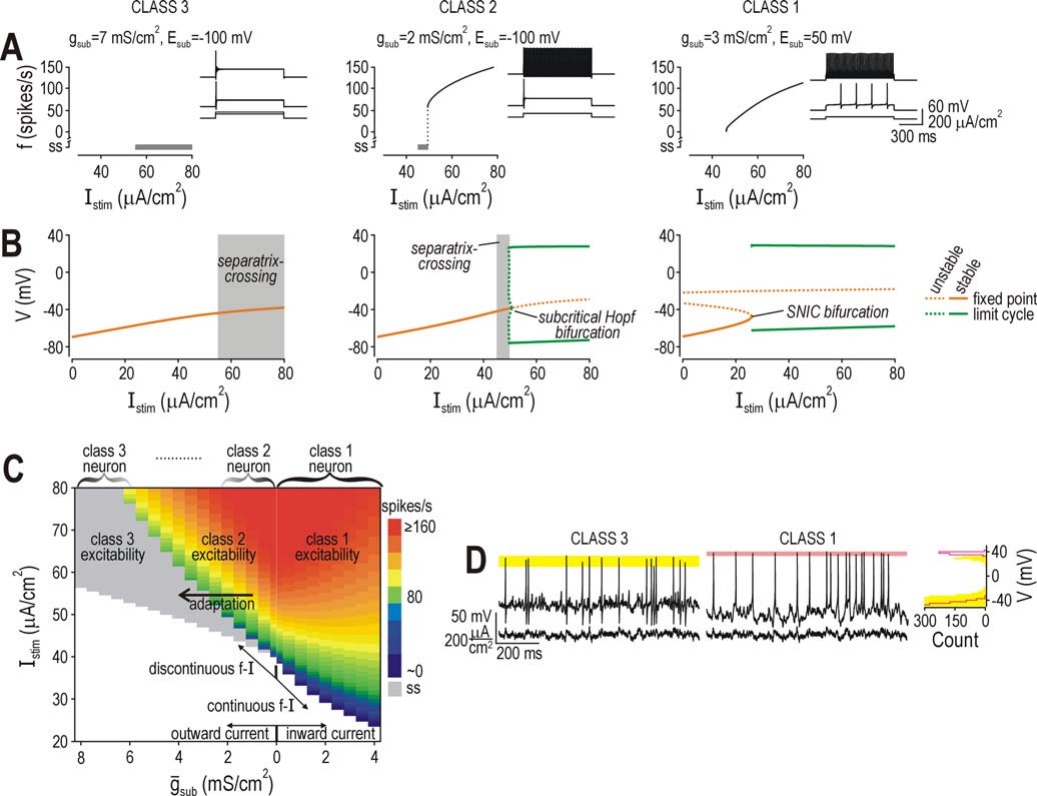
Sufficiency of oppositely directed subthrehsold currents to explain excitability. (A) Responses from 3D model described in Figure 4B. Without *I*_sub_, the model operated at the interface between class 1 and 2 excitability (see (C)). Adding an outward current (*E*_sub_ = −100 mV) produced class 2 or 3 excitability, with the latter becoming more predominant (i.e. over a wider range of *I*_stim_) as *ḡ*_sub_ was increased. Adding an inward current (*E*_sub_ = 50 mV) produced class 1 excitability. (B) Bifurcation diagrams show voltage at fixed point and at max/min of limit cycle as *I*_stim_ was increased. Class 1, 2, and 3 versions of the 3D models exhibited exactly the same spike initiating dynamics seen in class 1, 2 and 3 versions of the 2D models (compare with Figure 2B). (C) Firing rate (color) is plotted against *I*_stim_ and *ḡ*_sub_. These data are qualitatively identical to those for the 2D model (see Figure 1D) and indicate that direction and magnitude of *I*_sub_ are sufficient to explain different classes of excitability. The phasic-spiking that results from adaptation (see Figure 1C) can be understood in terms of slowly activating outward current (or inactivating inward current) causing a shift from class 2 to class 3 excitability. (D) As with the 2D model (Figure 3A), the class 3 version of the 3D model exhibited significantly greater spike amplitude variability than the class 1 version when driven by noisy stimulation (*p*<0.001, respectively; Kolmogorov-Smirnov test). σ_noise_ = 10 µA/cm^2^.

To demonstrate the sufficiency of subthreshold currents for determining excitability, we explicitly incorporated a subthreshold inward or outward current by adding an additional term for *I*_sub_ to the 2D model with *β*_w_ = −10 mV (see Equation 7); recall that the 2D model lies at the interface between class 1 and 2 excitability when *β*_w_ = −10 mV (see Figure 1D). Adding an inward current produced class 1 excitability, whereas adding an outward current produced class 2 or 3 excitability depending on the magnitude of *g*_sub_ (which is controlled by the maximal conductance, *ḡ*_sub_) (Figure 7A). With *β*_w_ = −13 mV (like the class 2 model in Figure 1B), the default 3D model was class 2; adding a subthreshold inward or outward current converted it to class 1 or 3, respectively (data not shown). The three classes of excitability can be readily identified from the bifurcation diagrams of the 3D model (Figure 7B; compare with 2D model in Figure 2B). Varying *ḡ*_sub_ affected the *f–I* curve in this 3D model in exactly the same manner as varying *β*_w_ in the 2D model (compare Figure 7C with Figure 1D). The transition between class 1 and 2 excitability occurred at *ḡ*_sub_ = 0 mS/cm^2^, although that value varied depending on *β*_w_ (see above). For a given value of *I*_stim_, class 1 and 2 excitability were mutually exclusive whereas class 2 and 3 excitability coexisted. Furthermore, the 3D model exhibited constant or variably sized spikes depending on whether the model was class 1 or 3, respectively (Figure 7D; compare with Figure 3A and 3B). This demonstrates the sufficiency of subthreshold inward and outward currents for producing class 1 and 3 excitability, respectively.

Thus, expression of distinct subthreshold currents accounts for the different classes of excitability observed amongst spinal lamina I neurons. But can other biophysical properties also account for differences in excitability? And, if so, do those properties confer the same or different spike initiating dynamics than those described above? In other words, can we generalize our biophysical explanation of excitability?

### Effects of Varying Other Parameters in the 2D Model

**Figure 8:**
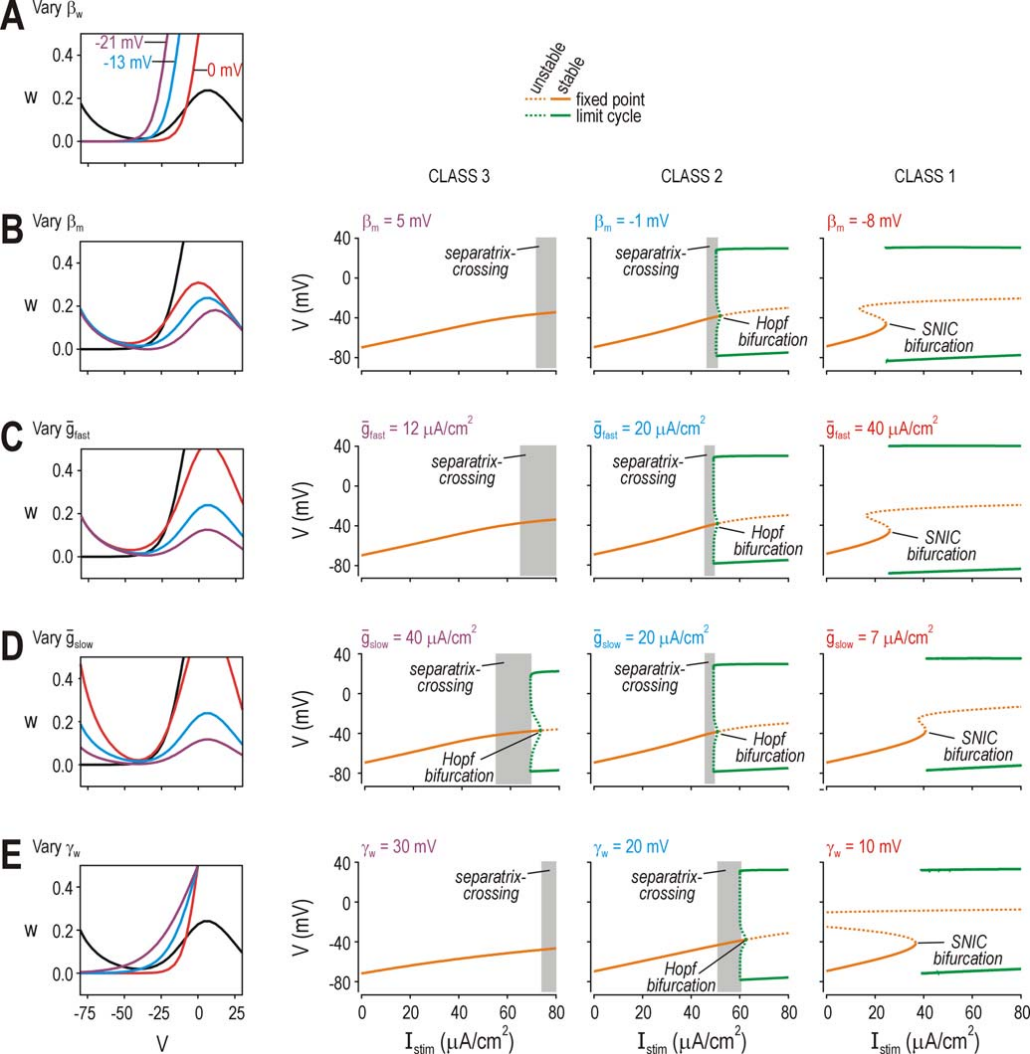
Common phase plane geometries associated with different parameter changes. (A) *β*_w_ controls positioning of the *w*-nullcline (i.e. voltage-dependency of *I*_slow_). For *β*_w_ = 0 mV, the nullclines intersect tangentially at rheobasic stimulation, which translates into an SNIC bifurcation. For *β*_w_ = −13 mV, the *w*-nullcline crosses the *V*-nullcline on its middle arm, which translates into a Hopf bifurcation. For *β*_w_ = −21 mV, the *w*-nullcline crosses the *V*-nullcline on its left arm, meaning spike initiation is limited to a QSC. See Figure 2B for corresponding bifurcation diagrams. Thus, spike initiating dynamics (and the resulting pattern of excitability) are directly related to phase plane geometry (i.e. how the nullclines intersect). (B) *β*_m_ controls positioning of the *V*-nullcline (i.e., voltage-dependency of *I*_fast_). Reducing *β*_m_ had the same effect on phase plane geometry as increasing *β*_w_. The predicted consequences for excitability are confirmed on the bifurcation diagrams. Like *I*_slow_, *I*_fast_ may comprise more than one current; therefore, differences in the voltage-dependency of the net fast current may reflect the expression of different fast currents rather than variation in the voltage-dependency of any one current (see Figure 4). For (B–E), *β*_w_ = −10 mV, *γ*_w_ = 13 mV, and all other parameters are as indicated in Methods unless otherwise stated. (C) Varying *ḡ*_fast_ changed the shape rather than positioning of the *V*-nullcline, but both had equivalent consequences for excitability. (D) Varying *ḡ*_slow_ also changed the shape of the *V*-nullcline, in a slightly different manner than *ḡ*_fast_, but with the same consequences for excitability. (E) Varying *γ*_w_, which controls the slope of the voltage-dependent activation curve for *I*_slow_, altered the *w*-nullcline, again, with predictable consequences for excitability. *β*_w_ = 0 mV.

We summarize here how spike initiating dynamics can be inferred from the phase plane geometry of the 2D model. We start by considering *β*_w_ (Figure 8A) since its effects on excitability have already been described in Figures 1 and 2. An SNIC bifurcation occurs when the *V* and *w*-nullclines intersect tangentially at rheobasic stimulation (*β*_w_>−10 mV). A Hopf bifurcation occurs when the *w*-nullcline crosses the *V*-nullcline on its middle arm (*β*_w_<−10 mV); although necessary, this is not strictly sufficient for the bifurcation (see below), but it is a close enough approximation for our demonstration. A QSC can occur when the *w*-nullcline crosses the *V*-nullcline on its left arm. Given this connection between phase plane geometry and spike initiating dynamics, one can predict the effects on excitability of changing the shape or relative positioning of the two nullclines regardless of which specific parameters are varied.

Moving the *V*-nullcline in one direction (via *β*_m_) should have the same effect as moving the *w*-nullcline in the opposite direction (via *β*_w_), which indeed it did (Figure 8B). The biophysical interpretation is straightforward: reducing *β*_m_ causes a hyperpolarizing shift in the voltage-dependency of *I*_fast_, causing *I*_fast_ to be more strongly activated by perithreshold depolarization and thus encouraging class 1 excitability. As explained in Figure 4 for *β*_w_, a change in *β*_m_ may reflect the contribution of a second fast current (inward or outward) with different voltage-dependency than the classic Na^+^ current comprising most of *I*_fast_. Increasing *ḡ*_fast_ in the 2D model without altering its voltage-dependency should also have an effect comparable to reducing *β*_m_, which indeed it did (Figure 8C). Thus, *β*_w_, *β*_m_, or *ḡ*_fast_ all affect phase plane geometry (i.e., how the nullclines intersect) in essentially the same way and with equivalent consequences for spike initiating dynamics. Although the specific biophysical mechanism is different in each case (voltage-dependency of *g*_slow_, voltage-dependency of *g*_fast_, or magnitude of *g*_fast_, respectively), the common outcome is a change in the balance of fast and slow currents near threshold.

It stands to reason, therefore, that reducing *ḡ*_slow_ (where *I*_slow_ is outward) should have effects comparable to increasing *ḡ*_fast_, which indeed it did (Figure 8D). Relatively large changes in *ḡ*_fast_ or *ḡ*_slow_ were required to convert excitability from class 1 to class 3, but one must consider that both of those net currents are most strongly activated at suprathreshold potentials. If spike initiating dynamics are dictated by currents at perithreshold potentials (see above), changes in maximal conductance should have small effects if the conductance is only marginally activated near threshold. By comparison, small changes in *ḡ*_sub_ were sufficient to alter spike initiating dynamics in the 3D model (see Figure 7) because *I*_sub_ was strongly activated at perithreshold potentials. Accordingly, reducing slope of the *w*-nullcline (by increasing *γ*_w_) extended the tail of the activation curve for *g*_slow_ so that *I*_slow_ was more strongly activated at perithreshold potentials; this predictably encouraged class 3 excitability (Figure 8E).

### Spike-Initiating Dynamics: Geometric Representation and Biophysical Explanation

Results in Figure 8 show that several different parameters can influence spike initiating dynamics (and, in turn, excitability), but there appear to be a limited number of phase plane geometries that result from varying those parameters. Other, more complex geometries are possible in higher dimensional systems, but the success of our 2D model in reproducing all of Hodgkin's classes attests that nonlinear interaction between two variables is sufficient to explain the patterns he described. Furthermore, “ultra-slow” processes like adaptation can, on a spike by spike basis, be treated as providing a constant current. Ultimately, spike generation is a two dimensional phenomenon requiring fast activation (positive feedback) to produce the rapid upstroke plus slower recovery (negative feedback) to produce the downstroke. Beyond shaping the spike waveform by their interaction at suprathreshold potentials, we explain here how these feedback processes interact at perithreshold potentials to control spike initiation.

**Figure 9:**
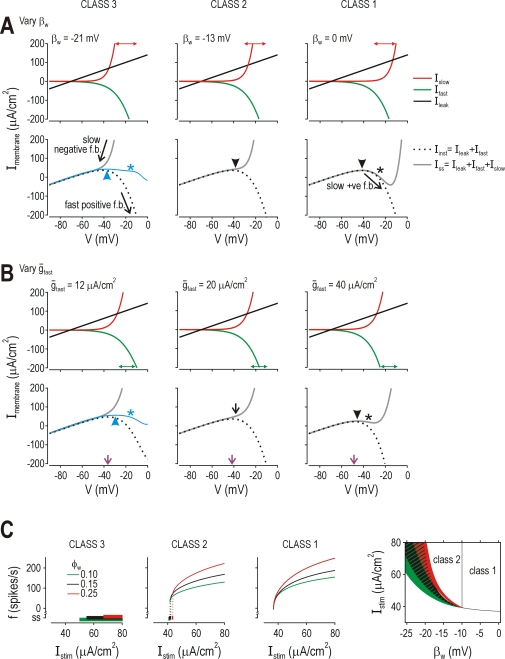
Competition between kinetically mismatched currents. (A) Top panels show individual currents in 2D model; bottom panels show how they combine to produce the instantaneous (*I*_inst_) and steady state (*I*_ss_) *I–V* curves. Double-headed arrows highlight effect of *β*_w_ on the voltage-dependency of *I*_slow_. Class 3 neuron: *I*_slow_ activates at lower *V* than *I*_fast_, meaning slow negative feedback keeps *V* from increasing high enough to initiate fast positive feedback *at steady state*. Fast positive feedback (that results in a spike) can be initiated only if the system is perturbed from steady state. Quasi-separatrix (blue) has a region of negative slope (*) indicating where net positive feedback occurs given the kinetic difference between fast and slow currents: positive feedback that activates rapidly can compete effectively with stronger negative feedback whose full activation is delayed by its slower kinetics. If *V* is forced rapidly past the blue arrowhead, fast positive feedback initiates a single spike before slow negative feedback catches up and forces the system back to its stable fixed point. Quasi-separatrix is plotted as the sum of all currents but with *I*_slow_ calculated as a function of *w* at the quasi-separatrix (see phase plane in Figure 2A) rather than at steady state and is shown here for *I*_stim_ = 60 µA/cm^2^. Class 2 neuron: *I*_slow_ and *I*_fast_ activate at roughly the same *V*. A Hopf bifurcation occurs at the point indicated by the arrow, where 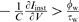 (see Results). This means that fast positive feedback exceeds slow negative feedback at steady state; as for class 3 neurons, this relies on positive feedback having fast kinetics since the net perithreshold current is still outward (i.e., steady state *I–V* curve is monotonic). Note that the slope of the steady-state *I–V* curve is less steep in the class 2 model than in the class 3 model. Class 1 neuron: *I*_slow_ activates at higher *V* than *I*_fast_, meaning slow negative feedback does not begin activating until after the spike is initiated. This gives a steady state *I*–*V* curve that is non-monotonic with a region of negative slope (*) near the apex of the instantaneous *I*–*V* curve. The SNIC bifurcation occurs when ∂*I*_ss_/∂*t* = 0 (arrowhead) because, at this voltage, *I*_fast_ counterbalances *I*_leak_ and any further depolarization will cause progressive activation of *I*_fast_. (B) Changing *ḡ*_fast_ in the 2D model had equivalent effects on the shape of the steady state *I–V* curves. Unlike in (A), voltage at the apex of the instantaneous *I*–*V* curve (purple arrows) changes as *ḡ*_fast_ is varied; in other words, the net current at perithreshold potentials can be modulated by changing fast currents (which directly impact voltage threshold) rather than by changing the amplitude or voltage-dependency of slow currents. This is consistent with results in Figure 8. (C) Speeding up the kinetics of *I*_slow_ impacts the onset of class 2 and 3 excitability. Compared with original model (*φ*_w_ = 0.15; black), increasing *φ*_w_ to 0.25 (red) increased *I*_stim_ required to cause a Hopf bifurcation or a QSC, but did not affect *I*_stim_ required to cause an SNIC bifurcation; reducing *φ*_w_ to 0.10 (green) had the opposite effect (summarized in right panel). Increasing *φ*_w_ also widened the discontinuity in the class 2 *f–I* curve and allowed class 2 and 3 neurons to achieve higher spiking rates with strong *I*_stim_ because of the faster recovery between spikes; reducing *φ*_w_ had the opposite effects.

Interpretation of the phase plane geometry can be formalized by doing local stability analysis near the fixed points ([27], see also chapter 11 in [28]). In class 3 neurons, 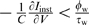 at the stable fixed point. This means, *at steady state*, that positive feedback is slower than the rate of negative feedback, *φ*_w_/*τ*_w_. Subthreshold activation of *I*_slow_ produces a steady state *I–V* curve that is monotonic and sufficiently steep near the apex of the instantaneous *I–V* curve that *V* is prohibited from rising high enough to strongly activate *I*_fast_ (Figure 9A, left). However, because the two feedback processes have different kinetics, a spike can be initiated if the system is perturbed from steady state: if *V* escapes high enough to activate *I*_fast_ (e.g., at the onset of an abrupt step in *I*_stim_), fast-activating inward current can overpower slow-activating outward current—the latter is stronger when fully activated, but can only partially activate (because of its slow kinetics) before a spike is inevitable. Through this mechanism, a single spike can be initiated before negative feedback forces the system back to its stable fixed point, hence class 3 excitability. Speeding up the kinetics of *I*_slow_ predictably allows *I*_slow_ to compete more effectively with *I*_fast_ (see below).

In class 2 neurons, 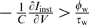 at the unstable fixed point, meaning positive feedback outpaces negative feedback, and repetitive spiking ensues. The steady state *I–V* curve (Figure 9A, center) is monotonic but less steep than in the class 3 model. Under these conditions, stimulation can force *V* high enough that fast-activating inward current competes with slow-activating outward current. As in the class 3 neuron (see above), it is crucial that *I*_fast_ activates more rapidly than *I*_slow_ in order for positive feedback to outrun negative feedback, since the latter would dominate and prohibit spiking if given enough time to fully activate. The difference from class 3 excitability is that fast positive feedback can outrun slow negative feedback with constant stimulation in a class 2 neuron; in the class 3 neuron, positive feedback can outrun negative feedback only during the stimulus transient.

In class 1 neurons, ∂*I*_ss_/∂*V* = 0 at the saddle-node, meaning the steady state *I–V* curve is non-monotonic with a local maximum above which depolarization activates net *inward* current at steady state (Figure 9A, right). The negative feedback responsible for spike repolarization only begins to activate at higher, suprathreshold potentials. Under these conditions, fast positive feedback has no slow negative feedback with which to compete at perithreshold potentials and the voltage trajectory can pass slowly through threshold, thereby producing slow spiking and a continuous *f–I* curve. That contrasts class 2 and 3 neurons in which activation of *I*_fast_ races subthreshold activation of *I*_slow_ to determine whether a spike is initiated. And although class 2 neurons can spike repetitively, they cannot maintain spiking below a critical frequency lest slow-activating outward current catch up with the fast-activating inward current.

Slope of the steady state *I–V* curve at perithreshold voltages (i.e., voltages near the apex of the instantaneous *I–V* curve) thus reveals the strength and direction of feedback with which *I*_fast_ must compete. Changing the direction and magnitude of *I*_sub_ in the 3D model had the same consequences on the steady state *I–V* curve (data not shown) as changing *β*_w_ in the 2D model. Changing other parameters in the 2D model, such as *ḡ*_Na_, also had similar effects (Figure 9B), which illustrates how the magnitude of slow current active at perithreshold voltages can be changed by modulating threshold rather than changing the voltage-dependency or density of the slow current itself (like in Figure 8B and 8C).

The *I–V* curve does not, however, provide information about the temporal features of the competition between kinetically mismatched currents. The relative kinetics of fast and slow currents are critical for class 2 and 3 excitability, whereas they are irrelevant for class 1 excitability since there is no competition (see above). To investigate this further, we changed the kinetics of *I*_slow_ by varying *φ*_w_ in the 2D model. Consistent with our dynamical explanations of spike initiation, speeding up *I*_slow_ increased the minimum stimulation required to produce class 2 or 3 excitability (especially the latter), whereas slowing down *I*_slow_ had the opposite effects; the minimum stimulation required to produce class 1 excitability was unaffected (Figure 9C). Increasing *φ*_z_ had the same effects in the 3D model (data not shown).

**Figure 10:**
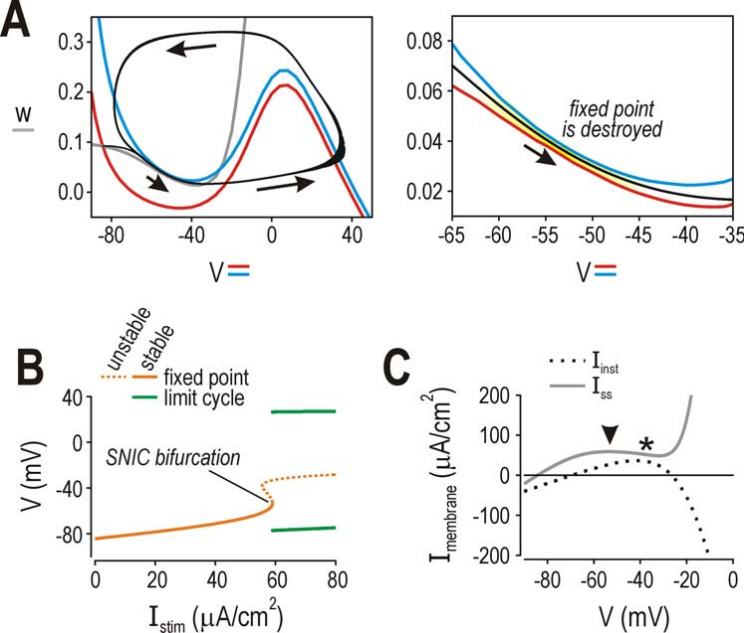
Depolarization-induced inactivation of a subthreshold outward current can also produce class 1 excitability. (A) Inactivation of an A-type K^+^ current by subthreshold depolarization should shift the balance of inward and outward currents the same way that depolarization-induced activation of an inward current does, and is therefore predicted to encourage class 1 excitability. To test this, we warped the *w*-nullcline to give it a region of negative slope at subthreshold potentials (see [55]); this was done by changing Equation 5 so that 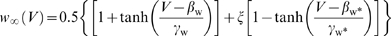 where *β*_w_ = −10 mV, *γ*_w_ = 10 mV, *β*_w*_ = −60 mV, *γ*_w*_ =  = 20 mV, and ξ = 0.1. Under these conditions, the *V*- and *w*-nullclines intersected tangentially at rheobasic stimulation. (B) This phase plane geometry resulted in an SNIC bifurcation and class 1 excitability, as predicted. (C) Inactivation of the A-type K^+^ current at subthrehsold potentials gave a region of negative slope on the steady state *I*–*V* curve that overlapped the apex of the instantaneous *I*–*V* curve.

If the balance of fast and slow currents at perithreshold potentials is the crucial determinant of excitability, then perithreshold *in*activation of a slow outward current (e.g., an A-type K^+^ current, *I*_K,A_) should encourage class 1 excitability the same way perithreshold activation of a slow inward current does. To test this, we incorporated *I*_K,A_ by warping the *w*-nullcline to create a 2D model similar to that of Wilson [29]. Rather than initiating spikes through a Hopf bifurcation, the *V*-nullcline intercepted the negatively sloping region of the *w*-nullcline tangentially (Figure 10A) and repetitive spiking was generated through an SNIC bifurcation (Figure 10B), which resulted in a continuous *f–I* curve (not shown) typical of class 1 excitability. Furthermore, inactivation of *I*_K,A_ introduced a region of negative slope into the steady state *I–V* curve that overlapped the apex of the instantaneous *I–V* curve (Figure 10C; compare with Figure 9). The same results were found if *I*_K,A_ was explicitly incorporated to produce a 3D model (data not shown). Thus, *I*_K,A_ increased rheobase but its slow inactivation as voltage passed through threshold amounted to a slow positive feedback process that encouraged class 1 excitability. The converse has been shown in medial superior olive neurons, where inactivation of *I*_Na_ encourages coincidence detection associated with class 3 excitability [30].

## Discussion

**Figure 11:**
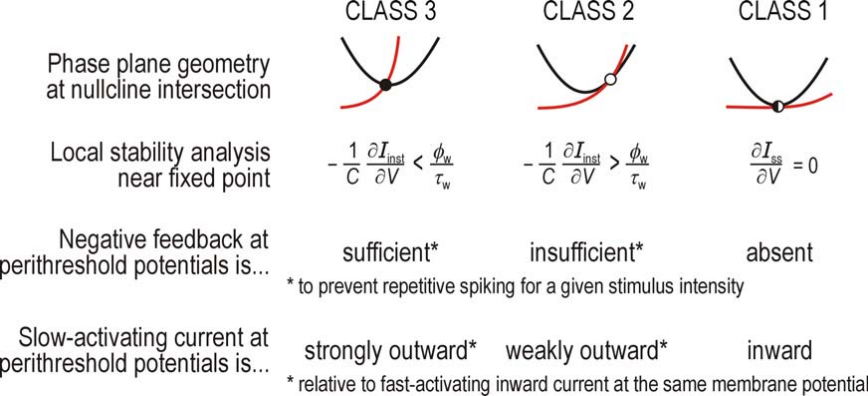
Summary of phase plane geometry and local stability analysis. Class 1 excitability results when slow-activating outward current is absent at voltages below threshold; inward current faces no competition and can drive arbitrarily slow spiking. Class 2 excitability results when outward current is activated at subthreshold voltages, but although net current is outward at steady state, fast-activating inward current ensures repetitive spiking above a critical frequency; spiking cannot be sustained below a rate that would allow enough time for slow-activating outward current to activate sufficiently that net current becomes outward during the interspike interval. Class 3 excitability results when outward current is sufficiently strong that repetitive spiking is prohibited despite fast-activating inward current; spike generation is only possible when the system is perturbed from steady state, as during a stimulus transient, during which fast-activating inward current initiates a spike before slow-activating outward current has an opportunity to counteract the positive feedback process.

Our results demonstrate that the three classes of excitability first described by Hodgkin [5] represent distinct outcomes in a nonlinear competition between fast and slow currents active at perithreshold potentials. We have experimentally confirmed (Figure 5) that spinal lamina I neurons express the currents predicted by our simulations (Figure 4) and that those currents are necessary (Figure 6) and sufficient (Figure 7) to produce the excitability exhibited by each cell type. Figure 11 summarizes the phase plane geometry associated with each class of excitability together with the results of local stability analysis near the fixed point, which explain how different spike initiating dynamics arise from a competition between fast and slow feedback processes. Voltage-dependent inward and outward currents mediate positive and negative feedback, respectively. Effects of changing the magnitude, voltage-dependency, or kinetics of those currents can be conceptualized in terms of how those parameter changes impact that competition; the consequences of such parameter changes for spike initiating dynamics are thus readily explained.

Our approach for uncovering the biophysical basis for Hodgkin's classification was to forward engineer a simple model in order to help reverse engineer complex neurons. The benefit of such an approach is that the model starts simple and is made only as complex as required to reproduce the phenomena of interest; extraneous details are thus excluded. Building a biologically realistic, high-dimensional model that exhibits one or another firing pattern is reasonably straightforward, but such a model almost certainly contains extraneous detail and may fail to provide greater insight than the experiments upon which it is based. The challenge when forward engineering simple models is that one may reproduce the phenomena of interest through a mechanism that is not the same as that used by real neurons; for example, a QSC produces a single-spiking pattern, but single-spiking neurons may use a completely different mechanism to produce the same result. This constitutes an inverse problem that requires careful consideration in order to validate the forward engineered model, as we demonstrated in Figure 3.

The forward engineering approach gave a model complex enough to reproduce each class of excitability yet simple enough for its spike initiating dynamics to be rigorously characterized using phase plane analysis. By expressing the problem geometrically, we were able to visualize and uncover the functional equivalence of changing different model parameters (Figure 8). As a result, our biophysical explanation of excitability is not specific to one set of neurons but, rather, should generalize to all neurons; for instance, spinal lamina I neurons exhibit different classes of excitability because they express different slow, subthreshold currents (Figures 5 and 6), but other neurons may achieve similar diversity through other mechanisms affecting spike initiation. Even a single neuron may exhibit different spike initiating dynamics depending on outside conditions such as the strength of background synaptic input [31]. According to our analysis, oppositely directed currents compete to determine spike initiation. Net current must be inward to sustain the depolarization necessary to initiate a spike (that much is obvious), but the balance between oppositely directed currents is not static. The importance of differences in the activation kinetics of the competing currents is far less obvious. In this regard, local stability analysis at the fixed point was critical for formalizing our explanation of the time-dependency of the competition, and for providing the insight that prompted us to test the effects of changing the relative kinetics of the competing currents (Figure 9C).

The mechanistic explanation of excitability afforded by quantitative analysis (i.e., phase plane analysis, local stability analysis, and bifurcation analysis) is precisely what is needed to make sense of the ever accumulating mass of experimental data. It provides an organizing framework for understanding which parameters are important and why, for instance, by explaining the functional equivalence of different biophysical changes (see Figure 8). By comparison, the reverse engineering approach, whereby the computational model is built with experimentally measured parameter values, does not typically provide results that can be so readily generalized.

According to our results, the direction, magnitude, and *kinetics* of currents activating or inactivating at perithreshold potentials influence the process of spike initiation. Previous studies have identified the competition between inward and outward currents as a critical determinant of excitability (e.g., [32]), but our results go further by explaining the importance of the temporal features of that competition. Conceptualizing spike initiation as a competition between fast and slow currents helps explain the shape of the *f–I* curve. If *I*_slow_ is absent or inward at perithreshold potentials, positive feedback mediated by *I*_fast_ faces no competition as it drives voltage slowly through threshold; a slow voltage trajectory between spikes means that the neuron can fire repetitively at low rates, thus producing the continuous *f–*I curve characteristic of class 1 excitability. If *I*_slow_ is outward at perithreshold potentials, then *I*_fast_ must compete with slow negative feedback. To compete successfully, *I*_fast_ must exploit its fast kinetics, which means driving voltage through threshold with sufficient rapidity that *I*_slow_ cannot catch up; a rapid voltage trajectory between spikes means that the neuron cannot fire repetitively at low rates, thus producing the discontinuous *f–I* curve characteristic of class 2 excitability. Whether *I*_slow_ is “strong enough” to prevent repetitive spiking altogether (thus producing class 3 excitability) depends on *I*_stim_, hence the diagonal border between class 2 and 3 excitability on Figures 1D and 7C; in contrast, the direction of feedback is independent of *I*_stim_, hence the vertical border between class 1 and 2 excitability on the same plots.

The adaptation observed in phasic-spiking neurons is also interesting insofar as it indicates that shifting the balance of fast and slow currents has important consequences for coding, and that a given neuron is not restricted to a unique spike initiation mechanism. Effects of activating an outward current or inactivating an inward current on ultra-slow time scales (across several ISIs) can be predicted from plots like Figure 7C: individual spikes are still generated through one of the three dynamical mechanisms we have described, but that mechanism can change over time according to dynamics of the ultra-slow process. Bursting, stuttering, and other interesting phenomena occur when ultra-slow processes interact with the fast-slow dynamics controlling spike initiation [7],[13],[33]. Understanding the relatively simple process of spike initiation will surely facilitate our understanding of how modulation of intrinsic properties impacts excitability and how more complex phenomena arise.

Although the dynamical bases for class 1 and 2 excitability have been established for some time [7], the connection between class 3 excitability and spike initiation through a QSC has not been made explicitly. The concept of spike initiation through a QSC was first proposed by FitzHugh [22],[23], who considered responses to brief pulses as well as to prolonged depolarizing and hyperpolarizing steps. However, Fitzhugh's explanation seems to have faded or, at best, is remembered in specific contexts such as post-inhibitory rebound excitation (e.g., [27]). Class 3 excitability has been reproduced in a Morris-Lecar-like model [34], but the spike initiating dynamics were not explored in that study. Izhikevich [13] accurately described class 3 excitability and ascribed it to *accommodation*, but this does not provide as satisfying a dynamical explanation as a QSC, which can accurately predict whether a spike will be initiated based on where the trajectory starts relative to the quasi-separatrix (see Figure 2). Indeed, the predictive power of a QSC (see Figure 3) attests to its utility and will hopefully lead to increased application of this concept within the field.

Experimental study of class 3 excitability has been neglected at least partly because of the mistaken assumption that all neurons displaying a single-spiking pattern are unhealthy (i.e., that the quality of the recording is poor). Indeed, an unhealthy neuron will often fail to spike repetitively, but many other indices of neuronal health (e.g., resting membrane potential) have proven that a single-spiking pattern is not synonymous with dysfunction. Indeed, “healthy” single-spiking neurons have been described not only in the superficial dorsal horn of the spinal cord [14],[15],[35], but also deeper in the spinal cord [36], as well as in dorsal root ganglia [37],[38], retina [39], amygdala [40], nucleus tractus solitarius [41], medial superior olive [42], medial nucleus of the trapezoid body [43], and anteroventral cochlear nucleus [44]. This list is not complete, at least in part because of the sampling bias explained above, but it nonetheless suggests that class 3 neurons are common within sensory pathways and certainly warrant increased investigation. Interestingly, mossy fiber boutons [45] and the axons of neocortical pyramidal neurons [46] also exhibit a single-spiking pattern when stimulated with prolonged depolarizing steps.

Class 3 excitability has been most extensively studied in the auditory system where the single- (or onset-) spiking pattern has been shown to result from a low-threshold K^+^ current [43],[47]. Svirskis et al. [30] have also shown that inactivation of Na^+^ current factors into the coding properties of those neurons, arguing that well-timed spikes are generated by rapid depolarizing input that minimizes activation of *I*_K,lt_ and inactivation of *I*_Na_; ideally, that rapid depolarization is preceded by hyperpolarizing input that primes the neuron by deactivating *I*_K,lt_ and deinactivating *I*_Na_. That biophysical explanation is consistent with our data. Single-spiking cells in the auditory system also exhibit variably sized spikes (e.g., [48]), which is also consistent with our results (see Figure 3A and 3B), as is the temperature-dependence of that variability [49]: in our model, the rate at which *V* changes relative to *w* is controlled by *φ*_w_ (see Equation 3) and is liable to vary with temperature [7], meaning temperature can influence spike amplitude in the model by changing how quickly trajectories peel away from the quasi-separatrix (data not shown). Based on these several lines of evidence, a QSC appears to be a robust explanation of the single-spiking pattern, not only for spinal lamina I neurons, but also for similar neurons elsewhere in the nervous system.

As explained in the Introduction, Hodgkin identified three classes of neurons based on phenomenological differences in their spiking pattern [5]. Subsequent work has linked that classification to differences in neural coding [6]–[12]. We have not formally investigated in this study how spike initiating dynamics impact neural coding, but increased understanding of spike initiation will facilitate future investigations into important issues such as spike-timing reliability. Indeed, our data (e.g., Figure 1) suggest that class 3 neurons are ideally suited to encode stimulus onset (timing) whereas class 1 neurons are better suited to encode stimulus intensity. Future work will focus on how spike initiating dynamics impact encoding of different stimulus features.

To conclude, Hodgkin's three classes of excitability result from different outcomes in a competition between fast and slow currents. The kinetic mismatch between currents is crucial for allowing single-spiking (class 3 excitability) or repetitive spiking faster than a critical frequency (class 2 excitability) despite the net steady state current being outward at threshold. Moreover, reproduction of qualitatively different spiking patterns in a 2D model emphasizes that rich dynamics are possible in simple systems based on their nonlinearities. Identifying functionally important nonlinearities and then determining how they are biologically implemented represents a powerful way of deciphering the functional significance of biophysical properties.

## Methods

### Slicing and Electrophysiology in Spinal Cord

All experiments were performed in accordance with regulations of the Canadian Council on Animal Care. Adult male Sprague Dawley rats were anesthetized with intraperitoneal injection of sodium pentobarbital (30 mg/kg) and perfused intracardially with ice-cold oxygenated (95% O_2_ and 5% CO_2_) sucrose-substituted artificial cerebrospinal fluid (S-ACSF) containing (in mM) 252 sucrose, 2.5 KCl, 2 CaCl_2_, 2 MgCl_2_, 10 glucose, 26 NaHCO_3_, 1.25 NaH_2_PO_4_, and 5 kynurenic acid; pH 7.35; 340–350 mOsm. The spinal cord was removed by hydraulic extrusion and sliced in the parasagittal plane as previously described [50]. Slices were stored at room temperature in normal oxygenated ACSF (126 mM NaCl instead of sucrose and without kynurenic acid; 300–310 mOsm) until recording.

Slices were transferred to a recording chamber constantly perfused at ∼2 ml/min with oxygenated (95% O_2_ and 5% CO_2_), room temperature ACSF. Lamina I neurons were visualized with gradient-contrast optics on a modified Zeiss Axioplan2 microscope (Oberkochen, Germany) and were patched on with pipettes filled with (in mM) 135 KMeSO_4_, 5 KCl, 10 HEPES, and 2 MgCl_2_, 4 ATP (Sigma, St Louis, MO), 0.4 GTP (Sigma); pH was adjusted to 7.2 with KOH and osmolarity ranged from 270–290 mOsm. Whole cell current clamp recordings were performed using an Axopatch 200B amplifier (Molecular Devices, Palo Alto, CA). Functional classification was determined from responses to 900 ms-long current steps [14] with pre-stimulus membrane potential adjusted to −60 mV by constant current injection in order to standardize across cells. Other stimuli included a noisy waveform generated through an Ornstein-Uhlenbeck process [51],
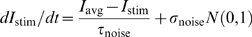
(1)where *N*(0,1) is a random number drawn from a Gaussian distribution with average 0 and unit variance, which is then adjusted according to size of the time step. Noise amplitude (*σ*_noise_) and filtering (*τ*_noise_) are reported in the text. DC offset (*I*_avg_) was adjusted to give roughly equivalent firing rates across different neurons.

Traces were low-passed filtered at 3–10 KHz and stored on videotape using a digital data recorder (VR-10B, Instrutech, Port Washington, NY). Recordings were later sampled at 10–20 KHz on a computer using Strathclyde Electrophysiology software (J. Dempster, Department of Physiology and Pharmacology, University of Strathclyde, Glasgow, UK).

### Computational Modeling

#### Two-dimensional model

Our starting model was derived from the Morris-Lecar model [7],[52] with a fast activation variable *V* and a slower recovery variable *w*. *V* represents voltage and controls instantaneous activation of fast currents (*I*_fast_); *w* is a function of voltage and controls activation of slower currents (*I*_slow_). Both *I*_fast_ and *I*_slow_ may comprise more than one current (see below); currents with similar kinetics were bundled together in order to create a low-dimensional model. The system is described by
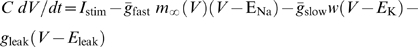
(2)

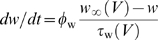
(3)

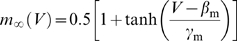
(4)

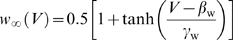
(5)

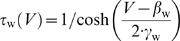
(6)Unless otherwise stated, *E*_Na_ = 50 mV, *E*_K_ = −100 mV, *E*_leak_ = −70 mV, *ḡ*_fast_ = 20 mS/cm^2^, *ḡ*_slow_ = 20 mS/cm^2^, *g*_leak_ = 2 mS/cm^2^, *φ*_w_ = 0.15, *C* = 2 µF/cm^2^, *β*_m_ = −1.2 mV, *γ*_m_ = 18 mV, *γ*_w_ = 10 mV, and *β*_w_ was varied as explained below.

This simple 2D model displayed each of Hodgkin's three classes of excitability but excluded details unnecessary for explaining the response properties of interest. Within that minimalist framework, we sought to isolate parameters sufficient to distinguish one class of excitability from another. Parameter values were found by manually varying them to produce a tonic- or single-spiking pattern. Once a set of parameters was found for each pattern, parameters were compared and adjusted to isolate those sufficient to explain each pattern. Varying *β*_w_ was found to be sufficient to convert the model between tonic-spiking (class 1 excitability) and single-spiking (class 3 excitability); varying other parameters including *ḡ*_fast_, *ḡ*_slow_, *β*_m_ or *γ*_w_ also affected excitability through the same geometrical changes associated with varying *β*_w_ (see Figure 8). Intermediate values of the aforementioned parameters consistently produced class 2 excitability.

#### Three-dimensional model

To make the model more biophysically realistic, we converted the 2D model into a 3D model (see Figure 4) by splitting *I*_slow_ into its component parts which include the delayed rectifier K^+^ current *I*_K,dr_ and a subthreshold current *I*_sub_ that is either inward or outward depending on *E*_sub_. Activation of *g*_K,dr_ and *g*_sub_ was controlled by *y* and *z*, respectively, so that
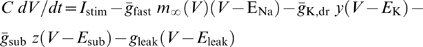
(7)

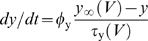
(8)

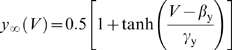
(9)

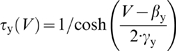
(10)

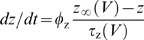
(11)

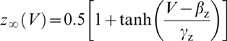
(12)

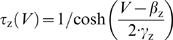
(13)
*I*_sub_ was either inward (*E*_sub_ = *E*_Na_ = 50 mV) or outward (*E*_sub_ = *E*_K_ = −100 mV) and *ḡ*_sub_ was varied. Kinetics of *I*_sub_ were adjusted to match experimental data so that *φ*_z_ = 0.5 for inward current and *φ*_z_ = 0.15 for outward current. The steady-state activation curve for *z* was the same for all models with *β*_z_ = −21 mV and *γ*_z_ = 15 mV. *φ*_y_ = 0.15, *β*_y_ = −10 mV, *γ*_y_ = 10 mV, and all other parameters were the same as in the 2D model.

#### Simulation methods

To stimulate the model, *I*_stim_ (in µA/cm^2^) was varied to produce steps, noise, or ramps comparable to stimuli used in experiments. Equations were integrated numerically in XPP [53] using the Euler method and a 0.1 ms time step. Phase plane and bifurcation analyses were performed according to standard procedures [7],[54]. Briefly, phase plane analysis involves plotting system variables relative to each other. Nullclines represent areas in phase space where a given variable remains constant. How the nullclines intersect (i.e., whether the intersection is stable or unstable) determines whether the system evolves towards a fixed point or towards a limit cycle (i.e., subthreshold membrane potential or repetitive spiking, respectively).

Nullclines were calculated in XPP [53]. For calculating a nullcline at time *t*, all variables not associated with the nullcline were held constant at their value at time *t*. Stability of fixed points was determined from the eigenvalues found by local stability analysis near the fixed point. A quasi-separatrix is distinct from a nullcline and was identified by the separation of flow on the phase plane (i.e., trajectories peal away to the right or left of the quasi-separatrix). The quasi-separatrix was plotted by integrating backward in time (−0.01 ms time step) from point * indicated on relevant figures; see Results for additional details.

In bifurcation analysis, *I*_stim_ was systematically varied to determine at what point the system qualitatively changes behavior (i.e., starts or stops spiking), which corresponds to a bifurcation. Whereas repetitive spiking is generated through a bifurcation, single-spiking generated through a QSC is not evident on a bifurcation diagram since the system's steady state has not changed. The stimulus range over which a QSC occurs is therefore indicated based on independent simulations.
